# Team leadership assessment after advanced life support courses comparing real teams vs. simulated teams

**DOI:** 10.3389/fpsyg.2022.1020124

**Published:** 2022-12-07

**Authors:** Sabine Nabecker, Sören Huwendiek, Fredy-Michel Roten, Lorenz Theiler, Robert Greif

**Affiliations:** ^1^Department of Anesthesiology and Pain Management, Sinai Health System, University of Toronto, Toronto, ON, Canada; ^2^Department of Anaesthesiology and Pain Medicine, Bern University Hospital, University of Bern, Bern, Switzerland; ^3^ERC ResearchNET, Niel, Belgium; ^4^Graduate School for Health Sciences (GHS), University of Bern, Bern, Switzerland; ^5^Department for Assessment and Evaluation, Institute for Medical Education, University of Bern, Bern, Switzerland; ^6^Department of Anaesthesia, Cantonal Hospital Aarau, Aarau, Switzerland; ^7^School of Medicine, Sigmund Freud University Vienna, Vienna, Austria

**Keywords:** education, assessment, CPR, advanced life support, ERC, human factors

## Abstract

**Aim:**

Effective team leadership is essential during cardiopulmonary resuscitation (CPR) and is taught during international advanced life support (ALS) courses. This study compared the judgement of team leadership during summative assessments after those courses using different validated assessment tools while comparing two different summative assessment methods.

**Methods:**

After ALS courses, twenty videos of simulated team assessments and 20 videos of real team assessments were evaluated and compared. Simulated team assessment used an instructor miming a whole team, whereas real team assessment used course participants as a team that acted on the team leader's commands. Three examiners individually evaluated each video on four different validated team leadership assessment tools and on the original European Resuscitation Council's (ERC) scenario test assessment form which does not assess leadership. The primary outcome was the average performance summary score between all three examiners for each assessment method.

**Results:**

The average performance summary score for each of the four assessment tools was significantly higher for real team assessments compared to simulated team assessments (all *p*-values < 0.01). The summary score of the ERC's scenario test assessment form was comparable between both assessment methods (*p* = 0.569), meaning that participants of both assessments performed equally.

**Conclusion:**

Team leadership performance is rated significantly higher in real team summative assessments after ALS courses compared to simulated team assessments by four leadership assessment tools but not by the standard ERC's scenario test assessment form. These results suggest that summative assessments in ALS courses should integrate real team assessments, and a new assessment tool including an assessment of leadership skills needs to be developed.

## Introduction

In Europe, the average survival rate at hospital discharge after out-of-hospital cardiac arrest ranges from 0 to 18% and in hospitals from 15 to 34% (Grasner et al., 2021; Perkins et al., 2021). This underlines how important proper cardiopulmonary resuscitation (CPR) education is to enable rescuers to improve patient outcomes.

Training is essential for lay persons, first responders (Marx et al., 2020; Nabecker et al., 2021a), and all healthcare providers (Smith et al., 2015; Greif et al., 2021) in small groups of up to 6 participants per instructor (Nabecker et al., 2021b) spaced over time (Yeung et al., 2020). Attendance in accredited advanced life support (ALS) courses improves patient outcomes (Lockey et al., 2018). The European Resuscitation Council (ERC) like other international resuscitation councils includes training in human factors especially team leadership (Greif et al., 2015; Kuzovlev et al., 2021; Soar et al., 2021) in their ALS courses. Nonetheless, the summative end-of-course assessments still largely focus on adherence to guidelines but do not assess team leadership and team members' success in task management.

Competency assessment practices of learners are different in various international ALS organizations. The ERC uses an assessment method where course participants are assessed as team leaders, while one instructor simulates a whole team. In courses run by the American Heart Association (AHA), a group of course participants acts as the team and the assessed course participant leads this team through a cardiac arrest scenario test.

Recently, we assessed course participants' and instructors' perceptions of how these two different assessment methods can test human factors. In short, real team assessment was preferred over simulated team assessment (Nabecker et al., 2022). This has been the first study comparing simulated vs. real team assessments. To the best of our knowledge, there are currently no other publications available on this topic. As different assessment methods are used during summative assessments in international CPR councils, it is important to establish which assessment method is better to test team leadership as an important human factor during cardiac arrest treatment.

The aim of this study was, therefore, to evaluate how well these two assessment methods allow instructors to test team leadership. We used four different validated assessment tools and the official ERC scenario test assessment form for the comparison of the two assessment methods.

The study results might trigger changes in the assessment approach of international resuscitation councils or provide evidence that a new assessment tool for ALS courses needs to be developed.

## Methods

### Study design

The videos of the summative assessments at the end of ALS courses were recorded between December 2017 and March 2019 and analyzed between March 2021 and September 2021. All course participants and instructors provided written informed consent for the study participation and the video recording.

### Setting

In the study year of 2017/2018, all fifth-year medical students at the University of Bern, Switzerland, participated in a mandatory 8-h immediate life support (ILS) course and all sixth-year medical students in a 16-h ALS course. All these courses were accredited ERC courses based on the 2015 ERC's resuscitation guidelines (Greif et al., 2015; Monsieurs et al., 2015). All instructors in these courses were ERC-certified ILS/ALS instructors. The study was registered at clinicaltrials.gov (NCT03412032) and was performed at the University of Bern, Switzerland.

At the end of each course, a mandatory summative assessment was held, which used the validated ERC cardiac arrest scenario tests (Ringsted et al., 2007). The courses were randomized with the “Research Randomizer” (https://www.randomizer.org) software to one of the two different assessment methods:

(1) Simulated team assessment: Only the assessed course participant and two instructors were in the assessment room. One instructor mimes a whole team. The miming instructor performs different team roles at once. The instructor does only act on the team leader's commands. The instructor does not introduce any standardized reactions. The assessed course participant acted as team leader and led this “Pro-forma” team through a cardiac arrest scenario test. This assessment method is currently the ERC standard test format.
(2) Real team assessment: Three additional course participants are with the assessed course participants in the assessment room. Only the team leader is assessed and the team members act as resuscitation team on the team leader's commands but are not allowed to advise the team leader on medical decisions. The assessed course participant acts as the team leader and leads this team through a cardiac arrest scenario test. The other course participants do not receive any further instructions, and they behave naturally. This assessment method approximates the current method used by the AHA.

All assessments were videotaped; videos were saved on a protected research server at the University of Bern.

### Study participants

Three certified ERC instructors (FMR, CS, and AH) watched a total of 40 randomly chosen videos (using the random selection function in Excel), 20 from the simulated team assessment and 20 from the real team assessment.

### Study measurements

The primary outcome parameter was defined as the average summary performance score for each assessment tool compared between both assessment methods. The null hypothesis of this study was that the summary performance scores of the included rating tools would be comparable between the real team and the simulated team assessments. The detailed results of each assessment tool used in this analysis are presented as Supplementary material.

### Study process

The three examiners rated team leadership and performance individually using four different validated team leadership and/or team performance checklists/tools as well as the original ERC ALS scenario test assessment forms. The intraclass correlation coefficient (ICC) was calculated for each rating tool. By using four different validated tools, we aimed to show the comparability of the assessment methods or the superiority of one assessment method. We did not provide cross-validation of the included rating tools. This was not the purpose of this study.

The four rating tools were:

(1) The checklist developed by Andersen et al. (2010): The checklist consists of 22 yes/no items targeting seven main topics, namely, initial therapy, continuous loops, information and supplementary therapy, spontaneous circulation, correction, maintenance algorithm, and technology. Each yes response was counted as 1 point, giving a maximum score of 22 points.
(2) The Concise Assessment of Leader Management instrument by Nadkarni et al. (2018): It asks the question whether the role of a team leader has been announced or not (yes = 1/no = 0), followed by 15 items assessing on a scale of rarely/sometimes/mostly/always the following areas: leadership, communication, team management, and medical management. For this analysis, we dichotomized the answers and counted a mostly or always response as 1 point, and a rarely or sometimes response as 0 points. Medical knowledge is assessed by free-text entries; therefore, we did not include this item in the current analysis. The last item is a global assessment of the team leader on a scale of below expected for level/as expected for level/above expectations for level/top 5%. Again, we dichotomized the responses. Each response for “As expected for level”, “above expectations for level”, or “top 5%” counted as 1 point, and “Below expected for level” as 0 points. This instrument, therefore, has a maximum score of 17 points.
(3) The Team Emergency Assessment Measure (TEAM) rating scale by Cooper et al. (2010): This scale consists of 11 items rated on a scale of 0 = never/hardly ever, 1 = seldom, 2 = about as often as not, 3 = often, and 4 = always/nearly always. Three different areas are covered, namely, leadership, teamwork, and task management. Again, we dichotomized the responses, and each response for “often” and “always/nearly always” was counted as 1 point and all others as 0 points. The last item on this scale is an overall rating on a Numeric Rating Scale from 1 to 10. Each response from 6 to 10 was counted as 1 point, and 5 and below as 0. Therefore, the maximum score for this rating scale was 12 points.
(4) The “leadership and behavior dimensions” are derived from a systematic review by Rosenman et al. (2015). We used the leadership dimension table as a yes/no checklist. The first item on this checklist was rated as one point if the response was yes either to leadership, defined in terms of clinical expertise, or to leadership, defined in terms of having a leader. There were additional three different areas with 37 items, namely, transition process, action process, and interpersonal skills. Each yes response counted as one point. This gives a maximum score of 38 points for this tool.
(5) The ERC ALS scenario test assessment forms are the official test forms used by the ERC. Items on this assessment form are derived from the ALS algorithms and do not include human factors or leadership items. The assessment form has 24 items rated on a scale from 1 to 4 (1 = outstanding, 2 = adequate, 3 = marginal, and 4 = insufficient). Each outstanding or adequate response was counted as 1 point, and all others as 0 points. A passing score was counted as 1 point, and not passing or retesting as 0 points. Therefore, the maximum score for the ERC ALS scenario test assessment form was 25 points.

### Study analysis

We did not perform a formal sample size calculation for this analysis as no data were available to base such a calculation (an extensive literature search in PubMed and Medline with the following search terms was performed (education, assessment, CPR, ALS, ERC, leadership, team, simulation, and human factor). Not a single publication resulted from that search comparing the two different assessment methods. However, we decided to include 20 videos from each of the two assessments as we assumed that more valuable information will not be derived by including more additional videos because the research question was whether the different assessment tools were able to assess team leadership but not to evaluate the individual course participant's performance.

Statistical analysis was performed using the STATA version 16.0 (StataCorp LT, Texas, USA) and IBM SPSS Statistics version 28.0.1.1 (IBM, New York, USA) software. The primary outcome was calculated by averaging each examiner's summary performance score for each assessment tool. The Mann–Whitney *U*-test was used to evaluate summary performance scores between assessment groups. Fisher's exact test was used to evaluate the detailed results presented in the supplemental material to this report. The ICC was calculated to compare inter-rater reliability between the 3 examiners. Data are presented as mean ± standard deviation (95% confidence interval) or value (percentage). A probability of <5% was considered statistically significant.

### Ethical considerations

The Cantonal Ethics Committee of Bern, Switzerland (Req-2017-00578, 7 August 2017) reviewed the study and judged that it does not fall under the Swiss Human Research Act of biomedical studies.

## Results

Table 1 shows the summary score for each checklist/tool used in this study and the ICC. All summary scores were significantly higher for the real team assessments compared to the simulated team assessments (*p* < 0.01). The summary score of the official ERC ALS scenario test assessment forms was comparable between both assessment methods (*p* = 0.569), meaning that participants of both assessments performed equally.

**Table 1:** Primary outcome parameter: summary score in each used checklist/tool between simulated team assessment and real team assessment.

	**Simulated team**	**Real team**	***p*-value^A^**
	**(*n* = 20)**	**(*n* = 20)**	
Checklist by Andersen et al. (2010)	12.2 ± 2.1 (11.2–13.2)	14.0 ± 2.0 (13.1–14.9)	<0.01
% of max. score of 22	55.5	63.6	
Intraclass correlation coefficient (ICC)	0.932	0.849	
Concise assessment of leader management by Nadkarni et al. (2018)	7.7 ± 2.9 (6.3–9.0)	10.5 ± 3.4 (8.9–12.1)	<0.01
% of max. score of 17	45.3	61.8	
Intraclass correlation coefficient (ICC)	0.819	0.801	
Team Emergency Assessment Measure (TEAM) by Cooper et al. (2010)	5.6 ± 1.7 (4.8–6.3)	9.5 ± 2.4 (8.4–10.6)	<0.01
% of max. score of 12	46.7	79.2	
Intraclass correlation coefficient (ICC)	0.444	0.611	
Leadership competencies by Rosenman et al. (2015)	15.4 ± 3.0 (14.0–16.8)	22.0 ± 4.6 (19.8–24.2)	<0.01
% of max. score of 38	40.5	57.9	
Intraclass correlation coefficient (ICC)	0.85	0.765	
Cardiac arrest simulation Test	17.9 ± 4.3	17.2 ± 3.8	0.569
	(15.9–19.8)	(15.4–18.9)	
% of max. score of 25	71.6	68.8	
Intraclass correlation coefficient (ICC)	0.877	0.896	

A Mann–Whitney U-test.

Scores are presented as mean ± standard deviation (95% confidence interval) and % of the total score.

In the Supplementary material to this report, we provide the detailed scores for each item and each checklist/tool included in this study. Hereafter, we showcase the most interesting findings.

In the checklist by Andersen et al. (2010), which was developed as a formative assessment tool for the measurement of performance of resuscitation teams with a maximum score of 22 points, the simulated team assessment received a mean of 12.2 points and the real team assessment received 14.0 points (*p* < 0.01, Table 1). It was possible to rate most items on this checklist with one or both assessment methods; however, there were 4 items that showed to be difficult to be rated with either assessment method. Difficulty in rating was discussed between the three examiners, and it was defined that some of the items simply could not be assessed with the rating tool used. Those were the use of cognitive aids or supplementary information and the correction of hyperventilation or defibrillation (compare Supplementary Table 1). For example, during an assessment, it is not allowed to use cognitive aids; therefore, this item could not be rated.

In the Concise Assessment of Leader Management instrument by Nadkarni et al. (2018) for formative assessment of team leader performance during pediatric resuscitations, a maximum score of 17 was achievable. The simulated team assessment reached a mean of 7.7 points, and the real team assessment reached a mean of 10.5 points (*p* < 0.01, Table 1). Most items were able to be scored; however, 3 items were difficult to be scored by either assessment method. Those were if a team leader was announced or not, reinforcement of closed-loop communication, and engagement of team members in decision-making (compare Supplementary Table 2).

The TEAM rating scale by Cooper et al. (2010), developed to rate medical emergency teamwork performance, had a maximum score of 12 points. The simulated team assessment scored a mean of 5.6 points, and the real team assessment 9.5 points (*p* < 0.01, Table 1). All items were able to be scored with both assessment methods (compare Supplementary Table 3).

The checklist derived from the leadership and behavior dimensions by Rosenman et al. (2015) allowed a maximum score of 38 points. The simulated team assessment scored a mean of 15.4 points and real team assessment scored 22.0 points (*p* < 0.01, Table 1). On this checklist, multiple items were difficult to be scored by either assessment, and those include team leader incorporates team member's suggestions, team leader briefs the team, team leader plans/decides how to do things, team leader debriefs the team/provides feedback/identifies areas for team improvement, team leader asks for help when needed, team leader notices changes in system/team environment, team leader identifies errors, team leader coaches/provides supervision as needed, team leader assists with conflict management/resolution, and team leader motivates and empowers team members (compare Supplementary Table 4).

On the official ERC ALS scenario test assessment forms, a maximum score of 25 points was achievable, the simulated team assessment received a mean of 17.9 points, and the real team assessment received 17.2 points (*p* = 0.569, Table 1). All items were able to be scored with both assessment methods. Supplementary Table 5 shows the detailed results of the scenario test assessment forms.

## Discussion

Assessed team leaders of real team assessments of ALS courses score significantly higher on four different validated assessment tools for team leadership performance than assessed team leaders of simulated team assessments. Therefore, the assessment method impacts how well-human factors can be assessed; simulated team assessments do not allow course participants to show leadership skills appropriately. This adds further evidence that a change in assessment practice to real team assessments might be beneficial to measure team leadership and performance of resuscitation teams if that is intended in the end-of-course assessment. Participants scored comparably in both assessment methods on the official ERC ALS scenario test assessment forms. Not surprising, this assessment tool does not include human factors.

In the current literature, there is only one study available comparing simulated vs. real team assessments. This study was performed by our research group and showed that instructors and participants favor real team assessments and judged the real team assessments mainly to be superior in assessing human factors. There is no other study available objectively evaluating real team vs. simulated team assessments.

Some items on the checklist by Andersen et al. (2010), developed as a formative assessment tool for team performance, were difficult to be scored for both assessment types, e.g., the use of cognitive aids and supplementary information, as well as the correction of hyperventilation and/or defibrillation. All those items are important during real cardiac arrests. The use of cognitive aids and supplementary information is, however, prohibited during summative assessments. The occurrence of hyperventilation and defibrillation errors during an assessment is highly circumstantial. It is not easy to standardize “poor” team member performance during an assessment that needs to be equal for all assessed participants. This checklist focuses mainly on team performance. Teamwork issues were identified as being most challenging by another study (Walsh et al., 2017). Training team membership is an integral part of all CPR courses. However, during the current final course assessments, only the team leader is assessed. Thoughts should be given as to whether the team should also be assessed during the final course assessment, which would be possible if real team assessments were used. There is currently no evidence available if formative assessments throughout the course could be used to replace the summative end-of-course assessment or at least the aspect of team membership (Greif et al., 2020). Therefore, future studies are necessary to establish if formative assessments can replace summative assessments for the assessment of leadership and other human factors.

On the Concise Assessment of Leader Management instrument by Nadkarni et al. (2018), again, some items were difficult to be scored as this tool was developed for formative assessments, not for summative assessments. The team leader was defined *a priori* as the person being assessed. The engagement of team members in decision-making was prohibited. Closed-loop communication seems difficult to be assessed, even though it should be possible to be judged at least by the real team assessment. Communication is a key component of human factors during cardiac arrests, and it has to be clear, brief, and empathetic and should provide a feedback loop (Jones et al., 2018; Ulmer et al., 2021). Literature shows that ongoing training on leadership principles improves communication (Hunziker et al., 2010, 2011; Lee et al., 2021). These results suggest a lack of competency of course participants in this specific human factor. However, if this is really the case, the implications for education and training need to be evaluated in further studies.

All items on the TEAM rating scale (Cooper et al., 2010) were able to be scored by either one of the assessment methods. This scale focuses on team leadership, teamwork, and team membership, and is widely used in ERC training courses without having established its scientific value in comparison with other scoring systems. There are certain aspects missing that other scores can map, e.g., situational awareness (Jones et al., 2018). Therefore, in the next step, it is important to develop a new assessment tool, which allows judging all major human factor aspects in conjunction with adherence to the current guidelines.

The checklist derived from leadership and behavioral dimensions by Rosenman et al. (2015) assesses team leadership comprehensively; however, there have been multiple items that none of the checklists were able to capture. Some are irrelevant or even prohibited during an assessment, e.g., the team leader incorporates team members' suggestions, or motivates or empowers team members. Other items are again highly dependent on circumstantial factors if they even occur, e.g., the team leader assists with conflict management/resolution. Those items are important to address during the debriefing of real cardiac arrests but are less relevant for assessments after ALS courses. Therefore, in a newly developed assessment tool, multiple items from this checklist can be omitted.

The official ERC ALS scenario test assessment form only asks for adherence to guidelines and does not test leadership skills. It is, therefore, not surprising that both assessment methods scored equally with it.

As participants of simulated team assessments scored significantly lower in human factor aspects than participants of real team assessments with the included checklist, but equal performance was found with the current ERC ALS scenario test assessment forms, we assume that this assessment method does not allow participants to show their full skill set in human factors. Therefore, future summative assessments in CPR courses should use real team assessments to account for that. None of the included assessment tools in this study seems to be perfect in scoring human factors targeted for team leadership. Therefore, future projects should consider creating a new and properly designed assessment tool specifically targeted for ALS course assessments that allow assessing team leadership as well as adherence to current resuscitation guidelines.

Limitations of this study are the single-center study design and the inclusion of only medical students as course participants, which might limit the generalizability of the results. All examiners were instructors at the same center. It is, therefore, possible that results would differ if different instructors from different areas or cultures had rated the participants' performance. We included three examiners, two men and one woman, with a similar extended experience as instructors to perform the video analysis. By using three examiners and adding the total scores together, differences in the rating of human factors were minimized. Another strength of this study is the execution of a randomized controlled trial.

## Conclusion

Participants of real team assessments of ALS courses score significantly higher on assessment tools evaluating human factors in comparison with simulated team assessments. The simulated team assessment prevents participants to show their learnt skills. These results support that real team assessment should be considered to be implemented in ALS courses. A new assessment tool should be created to incorporate human factor assessment as well as adherence to guidelines.

## Data availability statement

The datasets presented in this article are not readily available because the original contributions presented in this study are included in the article/Supplementary material, further inquiries can be directed to the corresponding author with a dedicated research question and local ethics committee approval. Requests to access the datasets should be directed to robert.greif@insel.ch.

## Ethics statement

Ethical approval was not required for the study on human participants in accordance with the local legislation and institutional requirements. The Cantonal Ethics Committee of Bern, Switzerland (Req-2017-00578, 07.08.2017) reviewed the study and judged that it does not fall under the Swiss Human Research Act of biomedical studies. All course participants and instructors provided written informed consent for the study participation and the video recording.

## Author contributions

SN had the idea of the study, performed data acquisition, data evaluation, first manuscript, and approved final manuscript. SH and LT contributed to data evaluation, significant contribution to manuscript, and approved final manuscript. F-MR contributed to data acquisition, significant contribution to manuscript, and approved final manuscript. RG performed data acquisition, data evaluation, significant contribution to manuscript, and approved final manuscript. All authors contributed to the article and approved the submitted version.
