# Supplementary material for: Team leadership assessment after advanced life support courses comparing real teams vs. simulated teams
Source: Front Psychol. 2022 Dec 7;13:1020124. doi: 10.3389/fpsyg.2022.1020124 (PMC9768360; doi:10.3389/fpsyg.2022.1020124)
Supplement: Supplementary file 1 [file Data_Sheet_1.docx]

**Supplementary Material to manuscript**

**Team leadership assessment after advanced life support courses comparing summative tests in real teams with simulated teams using five different assessment tools.**

**Table of Contents:**

Supplementary Table 1: Page 2

Supplementary Table 2: Page 4

Supplementary Table 3: Page 5

Supplementary Table 4: Page 6

Supplementary Table 5: Page 9

References: Page 12

**Supplementary Table 1: Checklist developed and published in 2010 by Andersen et al.^1^**

The checklist consists of 22 yes/no items. Each yes response was counted as 1 point, giving a maximum score of 22 points for this checklist.
Date is presented as number (%).

^A^Fisher’s exact test

|  | **Examiner 1** |  | **p-value^A^** | **Examiner 2** |  | **p-value^A^** | **Examiner 3** |  | **p-value^A^** |
| --- | --- | --- | --- | --- | --- | --- | --- | --- | --- |
|  | **Simulated team**  **(n=20)** | **Real team**  **(n=20)** |  | **Simulated team**  **(n=20)** | **Real team**  **(n=20)** |  | **Simulated team**  **(n=20)** | **Real team**  **(n=20)** |  |
| **BLS-established, yes** | 19 (95) | 20 (100) | 1.0 | 20 (100) | 20 (100) | NA | 20 (100) | 20 (100) | NA |
| **Immediate defibrillator** | 14 (70) | 17 (85) | 0.451 | 18 (90) | 18 (90) | 1.0 | 12 (60) | 18 (90) | 0.065 |
| **Monitoring** | 20 (100) | 20 (100) | NA | 20 (100) | 18 (90) | 0.487 | 20 (100) | 20 (100) | NA |
| **Time intervals** | 14 (70) | 19 (95) | 0.091 | 20 (100) | 20 (100) | NA | 2 (10) | 14 (70) | <0.01 |
| **Stop start compression** | 19 (95) | 20 (100) | 1.0 | 18 (90) | 20 (100) | 0.487 | 15 (75) | 19 (95) | 0.182 |
| **Medication safe** | 19 (95) | 20 (100) | 1.0 | 17 (85) | 18 (90) | 1.0 | 14 (79) | 17 (85) | 0.451 |
| **Change strategy after intubation** | 1 (5) | 5 (25) | 0.182 | 1 (5) | 8 (40) | 0.020 | 0 (0) | 7 (35) | <0.01 |
| **Change person 2 min** | 0 (0) | 15 (75) | <0.01 | 0 (0) | 17 (85) | <0.01 | 0 (0) | 16 (80) | <0.01 |
| **Cognitive aid** | 1 (5) | 0 (0) | 1.0 | 2 (10) | 0 (0) | 0.487 | 0 (0) | 0 (0) | NA |
| **Supplementary information** | 4 (20) | 5 (25) | 1.0 | 3 (15) | 2 (10) | 1.0 | 14 (70) | 17 (85) | 0.451 |
| **H and T evaluation** | 18 (90) | 16 (80) | 0.661 | 17 (85) | 16 (80) | 1.0 | 19 (95) | 16 (80) | 0.342 |
| **Reevaluation** | 17 (85) | 18 (90) | 1.0 | 19 (95) | 19 (95) | 1.0 | 13 (65) | 14 (70) | 1.0 |
| **Reevaluation ABCDE** | 17 (85) | 14 (70) | 0.451 | 19 (95) | 17 (85) | 0.605 | 18 (90) | 12 (60) | 0.065 |
| **Transfer monitoring** | 14 (70) | 10 (50) | 0.333 | 14 (70) | 14 (70) | 1.0 | 11 (55) | 9 (45) | 0.752 |
| **Correct unnec. Hands** | 13 (65) | 20 (100) | <0.01 | 12 (60) | 13 (65) | 1.0 | 0 (0) | 6 (30) | 0.020 |
| **Correct hyperventilation** | 0 (0) | 0 (0) | NA | 0 (0) | 0 (0) | NA | 0 (0) | 0 (0) | NA |
| **Correct defibrillation** | 0 (0) | 0 (0) | NA | 0 (0) | 0 (0) | NA | 0 (0) | 0 (0) | NA |
| **Algorithm defi** | 15 (75) | 7 (35) | 0.025 | 18 (90) | 19 (95) | 1.0 | 18 (90) | 18 (90) | 1.0 |
| **Algorithm medication** | 18 (90) | 14 (70) | 0.235 | 15 (75) | 15 (75) | 1.0 | 11 (55) | 13 (65) | 0.748 |
| **Algorithm pulse** | 17 (85) | 20 (100) | 0.231 | 16 (80) | 20 (100) | 0.106 | 17 (85) | 17 (85) | 1.0 |
| **Trouble shooting Defibr.** | 0 (0) | 0 (0) | NA | 0 (0) | 0 (0) | NA | 0 (0) | 0 (0) | NA |
| **Safe defibrillation** | 19 (95) | 20 (100) | 1.0 | 12 (60) | 14 (70) | 0.741 | 9 (45) | 19 (95) | <0.01 |

**Supplementary Table 2: Concise Assessment of Leader Management instrument developed, validated, and published in 2018 by Nadkarni et al.^2^**

This instrument consists of 1 yes/no question if the role of a team leader has been announced or not. A yes response was counted as 1 point. Then 15 items in 4 different areas follow which are assessed on a scale of rarely/sometimes/mostly/always. Each mostly or always response was counted as 1 point. On this tool the medical knowledge is assessed by a free text response and was therefore not included in this analysis. The last item was a global assessment of the team leaser on a scale below expected for level/ as expected for level/ above expectations for level/top5%. Each response for as expected for level or above was counted as 1 point. Therefore, the maximum score for this instrument was 17 points.
Datea are presented as number (%)

^A^Fisher’s exact test

| **Item** | **Examiner 1** |  | **p-value^A^** | **Examiner 2** |  | **p-value^A^** | **Examiner 3** |  | **p-value^A^** |
| --- | --- | --- | --- | --- | --- | --- | --- | --- | --- |
|  | **Simulated team**  **(n=20)** | **Real team**  **(n=20)** |  | **Simulated team**  **(n=20)** | **Real team**  **(n=20)** |  | **Simulated team**  **(n=20)** | **Real team**  **(n=20)** |  |
| **Announced Team leader** | 0 (0) | 2 (10) | 0.487 | 1 (5) | 4 (20) | 0.182 | 20 (100) | 20 (100) | NA |
| **Clear role leader** | 18 (90) | 19 (95) | 1.0 | 20 (100) | 19 (95) | 1.0 | 17 (85) | 16 (80) | 1.0 |
| **Style appropr.** | 17 (85) | 19 (95) | 0.605 | 19 (95) | 19 (95) | 1.0 | 11 (55) | 12 (60) | 1.0 |
| **Voice appropr.** | 16 (80) | 19 (95) | 0.342 | 17 (85) | 19 (95) | 0.605 | 16 (80) | 15 (75) | 1.0 |
| **Addresses people** | 1 (5) | 16 (80) | <0.01 | 1 (5) | 13 (65) | <0.01 | 0 (0) | 11 (55) | <0.01 |
| **Reinforces clc** | 0 (0) | 1 (5) | 1.00 | 2 (10) | 8 (40) | 0.065 | 1 (5) | 7 (35) | 0.044 |
| **Assisgn roles** | 4 (20) | 18 (90) | <0.01 | 16 (80) | 18 (90) | 0.661 | 2 (10) | 12 (60) | <0.01 |
| **Directs team** | 5 (25) | 18 (90) | <0.01 | 18 (90) | 19 (95) | 1.0 | 5 (25) | 12 (60) | 0.054 |
| **Balances workload** | 0 (0) | 18 (90) | <0.01 | 0 (0) | 18 (90) | <0.01 | 0 (0) | 10 (50) | <0.01 |
| **Engages member’s decis.** | 0 (0) | 1 (5) | 1.0 | 0 (0) | 3 (15) | 0.231 | 0 (0) | 3 (15) | 0.231 |
| **Summarizes status** | 9 (45) | 4 (20) | 0.176 | 6 (30) | 11 (55) | 0.200 | 9 (45) | 8 (40) | 1.0 |
| **Prioritizes tasks** | 13 (65) | 19 (95) | 0.044 | 18 (90) | 20 (100) | 0.487 | 12 (60) | 11 (55) | 1.0 |
| **Maintains global view** | 12 (60) | 15 (75) | 0.501 | 19 (95) | 20 (100) | 1.0 | 10 (50) | 11 (55) | 1.0 |
| **Periodically reassesses** | 11 (55) | 13 (65) | 0.748 | 16 (80) | 20 (100) | 0.106 | 17 (85) | 14 (70) | 0.451 |
| **States next steps** | 7 (35) | 4 (20) | 0.480 | 0 (0) | 5 (25) | 0.047 | 12 (60) | 8 (40) | 0.343 |
| **Limitations help** | 10 (50) | 4 (20) | 0.096 | 4 (20) | 8 (40) | 0.301 | 3 (15) | 4 (20) | 1.0 |
| **Global assessment** | 15 (75) | 14 (70) | 1.0 | 16 (80) | 18 (90) | 0.661 | 14 (70) | 11 (55) | 0.514 |

**Supplementary Table 3: The Team Emergency Assessment Measure (TEAM) rating scale developed and published in 2010 by Cooper et al.^3^**

The rating scale consists of 11 items rated on a scale of 0=never/hardly ever, 1=seldom, 2=about as often as not, 3=often, 4=always/nearly always. Each response as often and always/nearly always was counted as 1 point. The last item is an overall rating scale on a Numeric Rating Scale from 1-10. Each response from 6 and above was counted as 1 point.Therefore the maximum score for this rating scale was 12 points.
Data are presented as number (%).

^A^Fisher’s exact test

|  | **Examiner 1** |  | **p-value^A^** | **Examiner 2** |  | **p-value^A^** | **Examiner 3** |  | **p-value^A^** |
| --- | --- | --- | --- | --- | --- | --- | --- | --- | --- |
|  | **Simulated team**  **(n=20)** | **Real team**  **(n=20)** |  | **Simulated team**  **(n=20)** | **Real team**  **(n=20)** |  | **Simulated team**  **(n=20)** | **Real team**  **(n=20)** |  |
| **Team leader tells expectations** | 16 (80) | 19 (90) | 0.342 | 11 (55) | 11 (55) | 1.0 | 14 (70) | 13 (65) | 1.0 |
| **Team leader global perspective** | 14 (70) | 16 (80) | 0.716 | 19 (95) | 20 (100) | 1.0 | 14 (70) | 11 (55) | 0.514 |
| **Team communicated effectively** | 2 (10) | 15 (75) | <0.01 | 14 (70) | 19 (95) | 0.091 | 0 (0) | 12 (60) | <0.01 |
| **Timely manner** | 0 (0) | 20 (100) | <0.01 | 18 (90) | 20 (100) | 0.487 | 0 (0) | 15 (75) | <0.01 |
| **Composure control** | 0 (0) | 20 (100) | <0.01 | 17 (85) | 20 (100) | 0.231 | 9 (45) | 17 (85) | 0.019 |
| **Positive moral** | 3 (15) | 19 (95) | <0.01 | 18 (90) | 20 (100) | 0.487 | 0 (0) | 12 (60) | <0.01 |
| **Adaption to change** | 3 (15) | 19 (95) | <0.01 | 18 (90) | 20 (100) | 0.487 | 0 (0) | 13 (65) | <0.01 |
| **Reassessed situation** | 6 (30) | 18 (90) | <0.01 | 18 (90) | 20 (100) | 0.487 | 0 (0) | 13 (65) | <0.01 |
| **Aniticipated actions** | 0 (0) | 10 (50) | <0.01 | 3 (15) | 8 (40) | 0.155 | 0 (0) | 7 (35) | <0.01 |
| **Prioritises tasks** | 2 (10) | 20 (100) | <0.01 | 19 (95) | 20 (100) | 1.0 | 8 (40) | 13 (65) | 0.205 |
| **Followed guidelines** | 18 (90) | 18 (90) | 1.0 | 18 (90) | 18 (90) | 1.0 | 14 (70) | 12 (60) | 0.741 |
| **Global team rating** | 13 (65) | 16 (80) | 0.480 | 16 (80) | 17 (85) | 1.0 | 8 (40) | 11 (55) | 0.527 |

**Supplementary Table 4: The leadership and behavior dimensions derived from a systematic review of tools used to assess team leadership published by Rosenman et al in 2015.^4^**

We used the leadership dimensions table as a yes/no checklist. The initial not defined dimension was rated as one point if either the response was yes to leadership defined in terms of clinical expertise or in terms of having a leader. There were additional 37 items Each yes response was counted as 1 point, which results in a maximum score of 38 points.

Data are presented as number (%).

^A^Fisher’s exact test

| **Item**  **(Leadership/**  **Teamleader)** | **Examiner 1** |  | **p-value^A^** | **Examiner 2** |  | **p-value^A^** | **Examiner 3** |  | **p-value^A^** |
| --- | --- | --- | --- | --- | --- | --- | --- | --- | --- |
|  | **Simulated team**  **(n=20)** | **Real team**  **(n=20)** |  | **Simulated team**  **(n=20)** | **Real team**  **(n=20)** |  | **Simulated team**  **(n=20)** | **Real team**  **(n=20)** |  |
| **Leadership def. by having one** | 20 (100) | 20 (100) | NA | 20 (100) | 20 (100) | NA | 20 (100) | 20 (100) | NA |
| **Encourages team** | 0 (0) | 0 (0) | NA | 1 (5) | 7 (35) | 0.044 | 0 (0) | 6 (30) | 0.020 |
| **Incorporates suggestions** | 0 (0) | 3 (15) | 0.231 | 0 (0) | 1 (5) | 1.0 | 0 (0) | 5 (25) | 0.047 |
| **Keeps team informed** | 2 (10) | 3 (15) | 1.0 | 7 (35) | 13 (65) | 0.113 | 14 (70) | 12 (60) | 0.741 |
| **Confirms understanding** | 0 (0) | 1 (5) | 1.0 | 5 (25) | 11 (55) | 0.105 | 0 (0) | 1 (5) | 1.0 |
| **Reasseses** | 19 (95) | 20 (100) | 1.0 | 19 (95) | 20 (100) | 1.0 | 15 (75) | 17 (85) | 0.695 |
| **Briefs team** | 0 (0) | 2 (10) | 0.487 | 0 (0) | 6 (30) | 0.020 | 0 (0) | 3 (15) | 0.231 |
| **Asssign tasks** | 10 (50) | 17 (85) | 0.041 | 19 (95) | 19 (95) | 1.0 | 14 (70) | 20 (100) | 0.020 |
| **Sets expectations** | 0 (0) | 0 (0) | NA | 1 (5) | 9 (45) | <0.01 | 4 (20) | 5 (25) | 1.0 |
| **Uses guidelines** | 18 (90) | 19 (95) | 1.0 | 19 (95) | 19 (95) | 1.0 | 13 (65) | 14 (70) | 1.0 |
| **Plans next steps** | 19 (95) | 19 (95) | 1.0 | 20 (100) | 20 (100) | NA | 20 (100) | 20 (100) | NA |
| **Plans how to do sth.** | 3 (15) | 3 (15) | 1.0 | 1 (5) | 4 (20) | 0.342 | 20 (100) | 20 (100) | NA |
| **New plan if change** | 17 (85) | 18 (90) | 1.0 | 19 (95) | 20 (100) | 1.0 | 12 (60) | 12 (60) | 1.0 |
| **Prioritizes** | 13 (65) | 17 (85) | 0.273 | 19 (95) | 20 (100) | 1.0 | 7 (35) | 6 (30) | 1.0 |
| **Thinks ahead** | 7 (35) | 12 (60) | 0.205 | 3 (15) | 9 (45) | 0.082 | 4 (20) | 9 (45) | 0.176 |
| **Provides directions** | 13 (65) | 17 (85) | 0.273 | 15 (75) | 19 (95) | 0.182 | 9 (45) | 13 (65) | 0.341 |
| **Performs feedback** | 0 (0) | 0 (0) | NA | 0 (0) | 0 (0) | NA | 0 (0) | 0 (0) | NA |
| **Monitors progress** | 14 (70) | 17 (85) | 0.451 | 18 (90) | 19 (95) | 1.0 | 10 (50) | 7 (35) | 0.523 |
| **Notices changes** | 19 (95) | 19 (95) | 1.0 | 19 (95) | 20 (100) | 1.0 | 10 (50) | 11 (55) | 1.0 |
| **Asks for help** | 1 (5) | 0 (0) | 1.0 | 5 (25) | 10 (50) | 0.191 | 8 (40) | 6 (30) | 0.741 |
| **Notices system** | 0 (0) | 0 (0) | NA | 19 (95) | 19 (95) | 1.0 | 2 (10) | 13 (65) | <0.01 |
| **Facilitates team problem** | 0 (0) | 0 (0) | NA | 3 (15) | 11 (55) | 0.019 | 1 (5) | 7 (34) | 0.044 |
| **Remains hands off** | 18 (90) | 19 (95) | 1.0 | 20 (100) | 20 (100) | NA | 20 (100) | 17 (85) | 0.231 |
| **Engages in time management** | 6 (30) | 8 (40) | 0.741 | 16 (80) | 19 (95) | 0.342 | 0 (0) | 10 (50) | <0.01 |
| **Manages resources utiliz.** | 0 (0) | 4 (20) | 0.106 | 7 (35) | 18 (90) | <0.01 | 0 (0) | 13 (65) | <0.01 |
| **Manages progr.** | 2 (10) | 1 (5) | 1.0 | 17 (85) | 19 (95) | 0.605 | 1 (5) | 10 (50) | 0.003 |
| **Identifies error** | 1 (5) | 2 (10) | 1.0 | 2 (10) | 18 (90) | <0.01 | 10 (50) | 20 (100) | <0.001 |
| **Manages workload** | 1 (5) | 18 (90) | <0.01 | 0 (0) | 18 (90) | <0.01 | 0 (0) | 9 (45) | <0.01 |
| **Assists** | 0 (0) | 3 (15) | 0.231 | 1 (5) | 15 (75) | <0.01 | 0 (0) | 8 (40) | <0.01 |
| **Coaches as needed** | 0 (0) | 2 (10) | 0.487 | 0 (0) | 13 (65) | <0.01 | 0 (0) | 0 (0) | <0.01 |
| **Coordinate** | 10 (50) | 18 (90) | 0.014 | 16 (80) | 19 (95) | 0.342 | 1 (5) | 16 (80) | <0.01 |
| **Conflict management** | 0 (0) | 0 (0) | NA | 3 (15) | 3 (15) | 1.0 | 0 (0) | 0 (0) | NA |
| **Positive attitude** | 14 (70) | 19 (95) | 0.091 | 19 (95) | 19 (95) | 1.0 | 19 (95) | 19 (95) | 1.0 |
| **Treats with respect** | 10 (50) | 20 (100) | <0.01 | 20 (100) | 20 (100) | NA | 19 (95) | 20 (100) | 1.0 |
| **Balances authority** | 3 (15) | 11 (55) | 0.019 | 16 (80) | 20 (100) | 0.106 | 0 (0) | 8 (40) | <0.01 |
| **Copes with pressure** | 15 (75) | 17 (85) | 0.695 | 20 (100) | 20 (100) | NA | 12 (60) | 17 (85) | 0.155 |
| **Motivates & empowers** | 0 (0) | 1 (5) | 1.0 | 7 (35) | 18 (90) | <0.01 | 0 (0) | 3 (15) | 0.231 |
| **Communicates** | 5 (25) | 10 (50) | 0.191 | 11 (55) | 17 (85) | 0.082 | 2 (10) | 12 (60) | <0.01 |

**Supplementary Table 5:** ERC ALS scenario test assessment forms.

The assessment form has 24 items rated on a scale from 1-4, 1=outstanding, 2=adequate, 3=marginal, 4=insufficient. Each outstanding or adequate response was counted as 1 point. The last item on the assessment form is passing of the exam as yes/no answer. Each passing score was counted as 1 point. Therefore the maximum score was 25 points.

Data are presented as number (%).

^A^Fisher’s exact test

|  | **Examiner 1** |  | **p-value^A^** | **Examiner 2** |  | **p-value^A^** | **Examiner 3** |  | **p-value^A^** |
| --- | --- | --- | --- | --- | --- | --- | --- | --- | --- |
|  | **Simulated team**  **(n=20)** | **Real team**  **(n=20)** |  | **Simulated team**  **(n=20)** | **Real team**  **(n=20)** |  | **Simulated team**  **(n=20)** | **Real team**  **(n=20)** |  |
| **ABCDE** | 14 (70) | 18 (90) | 0.235 | 14 (70) | 15 (75) | 1.0 | 15 (75) | 12 (60) | 0.501 |
| **Oxygen/i.v.** | 17 (85) | 18 (90) | 1.0 | 14 (70) | 15 (75) | 1.0 | 15 (75) | 14 (70) | 1.0 |
| **Recognizes condition** | 14 (70) | 14 (70) | 1.0 | 12 (60) | 13 (65) | 1.0 | 10 (50) | 10 (50) | 1.0 |
| **Gives medication** | 8 (40) | 12 (60) | 0.343 | 5 (25) | 7 (35) | 0.731 | 8 (40) | 8 (40) | 1.0 |
| **Further medication** | 8 (40) | 5 (25) | 0.501 | 1 (5) | 5 (25) | 0.182 | 3 (15) | 5 (25) | 0.695 |
| **Other treatment** | 5 (25) | 4 (20) | 1.0 | 5 (25) | 3 (15) | 0.695 | 4 (20) | 2 (10) | 0.661 |
| **Recognizes arrest** | 16 (80) | 19 (95) | 0.342 | 19 (95) | 19 (95) | 1.0 | 18 (90) | 19 (95) | 1.0 |
| **Call for help** | 6 (30) | 11 (55) | 0.200 | 14 (70) | 12 (60) | 0.741 | 20 (100) | 19 (95) | 1.0 |
| **CRP for 2min** | 20 (100) | 20 (100) | NA | 20 (100) | 18 (90) | 0.487 | 20 (100) | 20 (100) | NA |
| **Airway** | 20 (100) | 20 (100) | NA | 9 (45) | 14 (70) | 0.200 | 19 (95) | 17 (85) | 0.231 |
| **Monitoring** | 20 (100) | 20 (1000 | NA | 19 (95) | 19 (95) | 1.0 | 14 (70) | 17 (85) | 0.451 |
| **Adrenalin** | 16 (80) | 15 (75) | 1.0 | 18 (90) | 15 (75) | 0.407 | 18 (90) | 15 (75) | 0.407 |
| **Reversible causes** | 18 (90) | 15 (75) | 0.407 | 15 (75) | 11 (55) | 0.320 | 14 (70) | 7 (35) | 0.056 |
| **Recognizes rythm** | 18 (90) | 19 (95) | 1.0 | 18 (90) | 19 (95) | 1.0 | 18 (90) | 19 (95) | 1.0 |
| **Defibrillation** | 15 (75) | 11 (55) | 0.320 | 18 (90) | 20 (100) | 0.487 | 19 (95) | 20 (100) | 1.0 |
| **CPR for 2min** | 19 (95) | 20 (100) | 1.0 | 18 (90) | 20 (100) | 0.487 | 20 (100) | 20 (100) | NA |
| **Recognizes rythm** | 16 (80) | 16 (80) | 1.0 | 13 (65) | 17 (85) | 0.273 | 16 (80) | 17 (85) | 1.0 |
| **Further adrenalin** | 13 (65) | 10 (50) | 0.523 | 10 (50) | 10 (50) | 1.0 | 5 (25) | 8 (40) | 0.501 |
| **Minimizes interruptions** | 13 (65) | 14 (70) | 1.0 | 13 (65) | 18 (90) | 0.127 | 11 (55) | 13 (65) | 0.748 |
| **Defibrillation** | 12 (60) | 5 (25) | 0.054 | 13 (65) | 15 (75) | 0.731 | 16 (80) | 8 (40) | 0.022 |
| **CRP for 2min** | 16 (80) | 14 (70) | 0.716 | 15 (75) | 17 (85) | 0.695 | 16 (80) | 14 (70) | 0.716 |
| **Recognizes rythm** | 16 (80) | 12 (60) | 0.301 | 18 (80) | 14 (70) | 0.235 | 19 (95) | 12 (60) | 0.020 |
| **Signs of life** | 16 (80) | 13 (65) | 0.480 | 19 (95) | 14 (70) | 0.091 | 17 (85) | 11 (55) | 0.082 |
| **Post resucitation** | 13 (65) | 7 (35) | 0.113 | 9 (45) | 10 (50) | 1.0 | 11 (55) | 9 (45) | 0.752 |
| **Result** | 15 (75) | 15 (75) | 1.0 | 15 (75) | 17 (85) | 0.695 | 17 (85) | 10 (50) | 0.041 |

**References**

1. Andersen PO, Jensen MK, Lippert A, et al. Development of a formative assessment tool for measurement of performance in multi-professional resuscitation teams. *Resuscitation* 2010;81(6):703-11. doi: 10.1016/j.resuscitation.2010.01.034

2. Nadkarni LD, Roskind CG, Auerbach MA, et al. The Development and Validation of a Concise Instrument for Formative Assessment of Team Leader Performance During Simulated Pediatric Resuscitations. *Simul Healthc* 2018;13(2):77-82. doi: 10.1097/SIH.0000000000000267

3. Cooper S, Cant R, Porter J, et al. Rating medical emergency teamwork performance: development of the Team Emergency Assessment Measure (TEAM). *Resuscitation* 2010;81(4):446-52. doi: 10.1016/j.resuscitation.2009.11.027

4. Rosenman ED, Ilgen JS, Shandro JR, et al. A Systematic Review of Tools Used to Assess Team Leadership in Health Care Action Teams. *Acad Med* 2015;90(10):1408-22. doi: 10.1097/ACM.0000000000000848
